# NMDA receptors antagonists alleviated the acute phase of traumatic brain injury

**DOI:** 10.22038/ijbms.2024.80887.17500

**Published:** 2025

**Authors:** Mehrdad Hajinejad, Ahmadreza Gharaeian Morshed, Abdolreza Narouiepour, Maryam Izadpanahi, Mohammad Mahdi Taheri, Mohammad Hossein Sadeghian, Fatemeh Forouzanfar, Sajad Sahab Negah

**Affiliations:** 1 Qaen Faculty of Medical Science, Birjand University of Medical Sciences, Birjand, Iran; 2 Department of Biology, Faculty of Science, Ferdowsi University of Mashhad, Mashhad, Iran; 3 Shefa Neuroscience Research Center, Khatam Alanbia Hospital, Tehran, Iran; 4 Department of Anatomy, School of Medicine, Iranshahr University of Medical Sciences, Iranshahr, Iran; 5 Neuroscience Research Center, Mashhad University of Medical Sciences, Mashhad, Iran; 6 Department of Neuroscience, Faculty of Medicine, Mashhad University of Medical Sciences, Mashhad, Iran; 7 Department of Pharmaceutical Biomaterials, Faculty of Pharmacy, Tehran University of Medical Sciences, Tehran, Iran; 8 Department of Forensic Medicine, School of Medicine, Tehran University of Medical Sciences, Tehran, Iran

**Keywords:** Ketamine, Memantine, Neuroinflammation, NMDA receptor antagonist, Oxidative stress, Traumatic brain injury

## Abstract

**Objective(s):**

Traumatic brain injury (TBI) is a significant cause of mortality and disability worldwide. TBI has been associated with factors such as oxidative stress, neuroinflammation, and apoptosis, which are believed to be mediated by the N-methyl-D-aspartate (NMDA)-type glutamate receptor. Two NMDA receptor antagonists, ketamine and memantine, have shown potential in mitigating the pathophysiological effects of TBI.

**Materials and Methods:**

To conduct the study, a controlled cortical impact model was used to induce TBI in rats. The rats with TBI were then divided into three groups: a group receiving only TBI, a group receiving TBI along with memantine, and a group receiving TBI along with ketamine. After 24 hr, the levels of oxidative stress markers (such as SOD, MDA, and total thiol) in the brain tissue were measured. Immunohistochemical staining was also performed seven days after TBI to assess the activation of glial cells and the TLR-4/NF-κB neuroinflammatory pathway.

**Results:**

The results indicated that treatment with memantine led to a reduction in MDA levels and an increase in SOD and total thiol levels. Memantine also decreased astrogliosis and down-regulated the TLR-4/NF-κB pathway. On the other hand, ketamine increased the levels of anti-oxidant markers but did not significantly affect the MDA level. Additionally, ketamine decreased the expression of NF-κB seven days after TBI.

**Conclusion:**

The findings suggest that NMDA receptor antagonists, such as ketamine and memantine, may have therapeutic effects on TBI by inhibiting oxidative stress and inflammatory responses.

## Introduction

Traumatic brain injury (TBI) is a significant global health concern, affecting millions of individuals yearly and leading to disabilities and fatalities (1, 2). TBI can result from various external forces on the head, such as blows, penetrations, or head acceleration/deceleration (3).

Two phases characterize the pathophysiology of TBI: the primary injury, which is the initial mechanical trauma causing strains on neural and vascular tissues, increased intracranial pressure, and bleeding (3), and the delayed secondary injury, which can occur within hours to weeks after the injury and involves cellular and molecular events like imbalanced calcium homeostasis, glutamate toxicity, mitochondrial dysfunction, oxidative stress, brain edema, and neuroinflammation (4, 5).

Neuroinflammation is a critical secondary response of the brain to insults like trauma, activating and recruiting various immune cells (4, 6). Microglia and astrocytes, two types of glial cells, play essential roles in the noninflammatory response by releasing proinflammatory mediators (7, 8). Secondary injury and neuroinflammation can lead to delayed and prolonged neurological disorders (9). Interacting during this secondary response is crucial to prevent irreversible damage (10).

In TBI, the accumulation of glutamate in the central nervous system can disrupt cellular function and promote cell death. Excessive activation of N-methyl-D-aspartate (NMDA) receptors by glutamate leads to an oxidative stress imbalance and inflammation, resulting in tissue damage (11-15). NMDA receptors are crucial for brain functioning, including memory and neuronal plasticity (16). The overstimulation of the NMDA receptors causes an influx of sodium and calcium ions, exacerbating the injury and leading to neuronal dysfunction and death. Modulating NMDA receptors has gained attention in recent research (17). Pharmacological interventions to inhibit glutamate receptors, such as memantine and ketamine, may offer neuroprotective effects by blocking NMDA receptors and preventing calcium influx (18).

Ketamine, besides its effect on NMDA receptors, provides hemodynamic stability by inhibiting NO synthesis. It also targets hippocampal cell proliferation, improving learning and behavioral outcomes after brain injury (19). Memantine exhibits anti-oxidative and anti-inflammatory effects against neuronal damage by reducing malondialdehyde (MDA (levels and enhancing the expression of nuclear factor-kappa B (NF-κB), Tumor Necrosis Factor-alpha (TNF-α), and Interleukin-1 beta (IL-1β) (20, 21). Given these effects, this study aims to investigate and compare the effects of memantine and ketamine on the main mechanisms involved in TBI pathophysiology.

## Materials and Methods

***Animals***

This study utilized 33 male Wistar rats weighing 200–220 g, obtained from the Animal Core Facility at the Mashhad University of Medical Sciences in Iran. The rats were housed under standard conditions with a temperature of 23±2 °C, 65±10% humidity, and a 12-hour light/12-hour dark cycle. The Animal Care Ethical Committee of Mashhad University of Medical Sciences approved and conducted the research procedures according to institutional guidelines.

***TBI model and experimental procedure***

To induce a controlled cortical impact (CCI) model of TBI, the rats were anesthetized using an intraperitoneal (IP) injection of sodium thiopental (5 mg/kg). The animals were secured in a stereotaxic frame (World Precision Instruments, USA), and the scalp was shaved and cut open to expose the skull bone. A craniotomy was performed at AP = 2 mm and ML = − 1 mm, removing the dura matter. The CCI model was induced with an impactor (impact velocity, 0.1 m/sec; impact duration, 0.2 sec; impact depth, 1.5 mm), after which the skin was carefully closed, and the animals were returned to their standard cages. 

After induction of injury, rats were randomly allocated into three groups (n=11) for the experimental procedures: TBI (no treatment), TBI+ Ketamine (Intraperitoneal (IP) administration of ketamine for seven days after CCI model, with a high dose of 20 mg/kg immediately after injury and a low dose of 10 mg/kg for six consecutive days), and TBI+ Memantine (IP administration of Memantine for seven days after CCI model, with a high dose of 5 mg/kg immediately after injury and a low dose of 1 mg/kg for six consecutive days).

***Evaluation of oxidative stress***

After 24 hr of injury, the levels of oxidative stress in the brain tissue were assessed. Six animals were euthanized using IP administration of ketamine (80 mg/kg) and xylazine (10 mg/kg). The brain samples were collected and homogenized using a phosphate buffer solution (PBS) with a pH of 7.4. The homogenates were then centrifuged at 1500 rpm for 10 min. The resulting supernatant was used to measure the levels of MDA, total thiol groups, and the activity of superoxide dismutase (SOD).

***MDA assay***

To determine the concentration of MDA, a marker of lipid peroxidation, the brain tissue samples were mixed with a working solution composed of 15% w/v trichloroacetic acid, 0.375% w/v thiobarbituric acid, and 0.25 M hydrochloric acid. The resulting mixture was then heated in a water bath for 45 min to form a pink complex with a peak absorbance of 535 nm. After centrifugation, the absorbance of the supernatant was measured at 535 nm using a spectrophotometer (22). The concentration of MDA was calculated using the formula C (M) = A/1.65×10^5^, where C represents the concentration and A denotes the absorbance.

***Evaluation of SOD activity***

The researchers used the method proposed by Madesh and Balasubramanian to measure the activity of superoxide dismutase (SOD) (23). The technique involves generating superoxide by autoxidation of pyrogallol and inhibiting the superoxide-dependent reduction of 3-4,5-dimethyl-thiazol-2-yl-2,5-diphenyl tetrazolium bromide (MTT) to formazan. The procedure involved adding 60 µl of sample and 15 µl of pyrogallol to each well. Next, 6 µl of MTT was added to each well, except for the standard well, which received 6 µl of BPS. The reaction was terminated with dimethyl sulfoxide (DMSO), and the plate was measured at a wavelength of 570 nm using a microplate reader.

***Measurement of total thiol content***

The thiol family is a versatile, robust defense system against reactive oxygen species (ROS) production (24). The Ellman method was utilized to assess the total thiol content, which is also known as sulfhydryl groups, using 5,5′-dithiobis (2-nitrobenzoic acid) (DTNB) (25). The absorbance of a 50 µl sample was recorded at 412 nm against tris-EDTA buffer (pH = 8.2) (A1). Then, 20 µl of DTNB (10 M) was added to the solution, and the mixture was incubated at room temperature. After 15 min, the absorbance of the sample was measured again (A2). The following formula was utilized to determine the total thiol content: Total thiol concentration (mM) = (A2-A1-B) × 1.07/ (0.05 × 13.6).

***Immunohistochemistry evaluation***

After inducing deep anesthesia, brain tissue samples were extracted from animals (n=5). The samples were then fixed in 10% buffered formaldehyde for five days. After dehydration, the samples were embedded in paraffin and sectioned into 5 µm slices. The sections were rehydrated and subjected to antigen retrieval by boiling in PBS with a pH of 6.0. Endogenous peroxidase activity was blocked using a 3% hydrogen peroxide (H_2_O_2_) solution. The sections were incubated in 10% normal goat serum (NGS) for 60 min to block any undesirable background staining. Various primary antibodies were then applied, including Rabbit anti-Glial Fibrillary Acidic Protein (GFAP), which acts as an astrocyte marker (1:2000; ab7260, Abcam, USA), Rabbit anti-Ionized calcium-binding adapter molecule 1 (Iba1), used as a microglia marker (1:1000; LKG5732, Wako, USA), Rabbit anti-Toll-like receptor 4 (TLR4) antibody (1:500; ab217274, Abcam, USA), Rabbit anti-nuclear factor-kappa B (NF-κB) p65 antibody (1:1000; ab16502, Abcam, USA) .After applying the primary antibodies, the sections were refrigerated overnight at 4 °C. The next day, the sections were incubated with a horseradish-peroxidase (HRP)-conjugated anti-rabbit secondary antibody (1:1000; ab6721, Abcam, USA) at room temperature for one hour. The treated sections were visualized using 3,3’-diaminobenzidine (DAB) and were subsequently counterstained with hematoxylin before being mounted onto glass slides. To quantify the number of positive cells post-immunohistochemical staining, a standard lens was used across all groups and animals for each marker at the site adjacent to the lesion (situated at the cavity’s edge). Specifically, a 20X lens was used to count GFAP and Iba-1 positive cells, while a more powerful 40X lens was used to count TLR-4 and NF- κB positive cells.

***Statistical analysis***

The data are presented as mean ± standard error of the mean (SD). To perform statistical comparisons, Graph Pad Prism version 8 was used. One-way analysis of variance (ANOVA) was conducted, followed by Tukey’s *post hoc* test to calculate *P*-values for the statistical comparisons. Differences were considered statistically significant if the *P*-value was less than 0.05.

## Results

***Effects of ketamine and memantine on TBI-induced oxidative stress***

**Figure 1:**
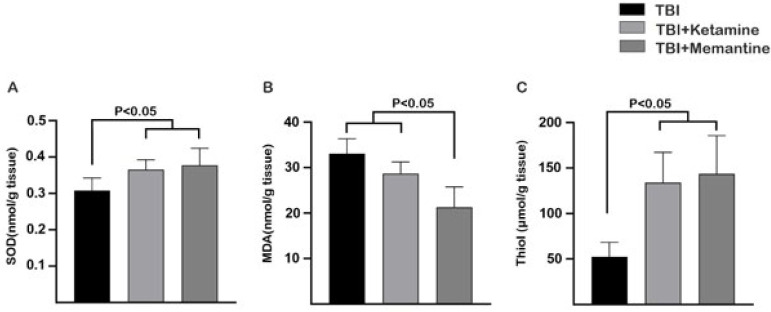
Oxidative stress assay 24 hr after rat TBI model

This study examined the potential therapeutic effects of ketamine and memantine on oxidative stress by measuring various anti-oxidant factors, including SOD, MDA, and total thiol levels in brain tissue. The results showed that ketamine significantly increased the levels of SOD and thiol 24 hr after injury (Figure 1A and C) but did not significantly reduce the MDA level compared to the TBI group (Figure 1B). On the other hand, treatment with memantine increased the levels of SOD and thiol compared to the TBI group (Figure 1A and C) while significantly decreasing the brain tissue levels of MDA compared to both the ketamine and TBI groups.

***Effects of ketamine and memantine on glial cells activation***

**Figure 2:**
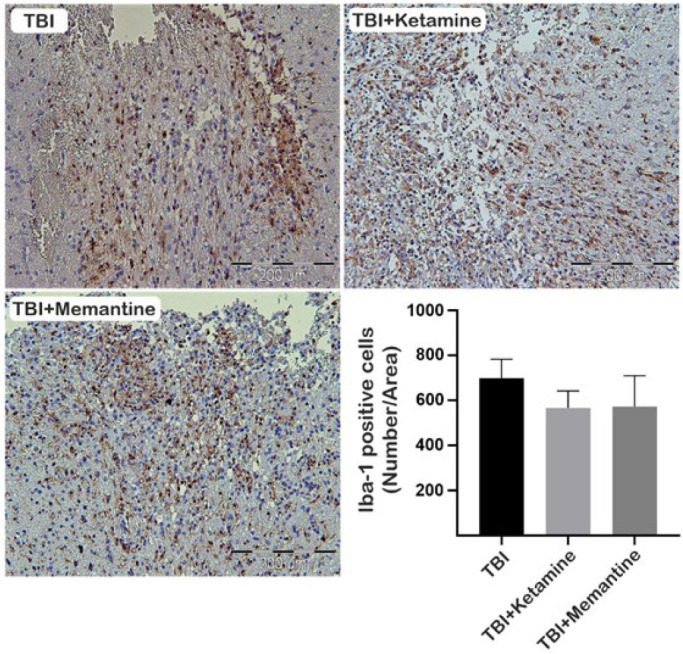
Effect of ketamine and memantine on microglia activation following TBI in rats

**Figure 3:**
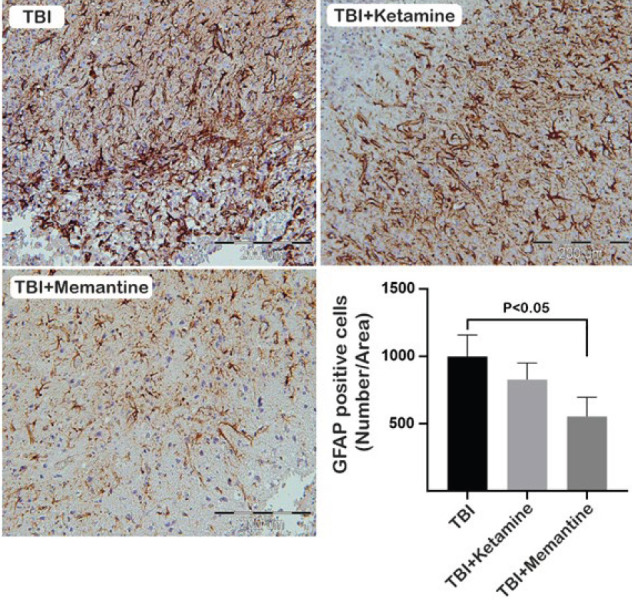
Effect of ketamine and memantine on astrocyte activation following TBI in rats

Activation of glial cells is a rapid and essential mechanism underlying the damage induced by TBI, with astrocyte and microglia reactivity measured through up-regulation of Iba-1 and GFAP immunohistochemical labeling, respectively (26, 27). The expression of Iba-1 protein, a microglia marker, was found to increase in brain tissue after TBI, but treatment with ketamine and memantine did not significantly decrease this expression seven days after injury (Figure 2). As shown in Figure 3, analysis of brain tissue sections also revealed that treatment with ketamine did not significantly decrease GFAP-positive cells compared to the TBI group. Still, therapy with memantine significantly reduced the number of GFAP-positive cells seven days after injury.

***Effects of ketamine and memantine on TLR4/NF-*** ***κB*** ***pathway***

**Figure 4:**
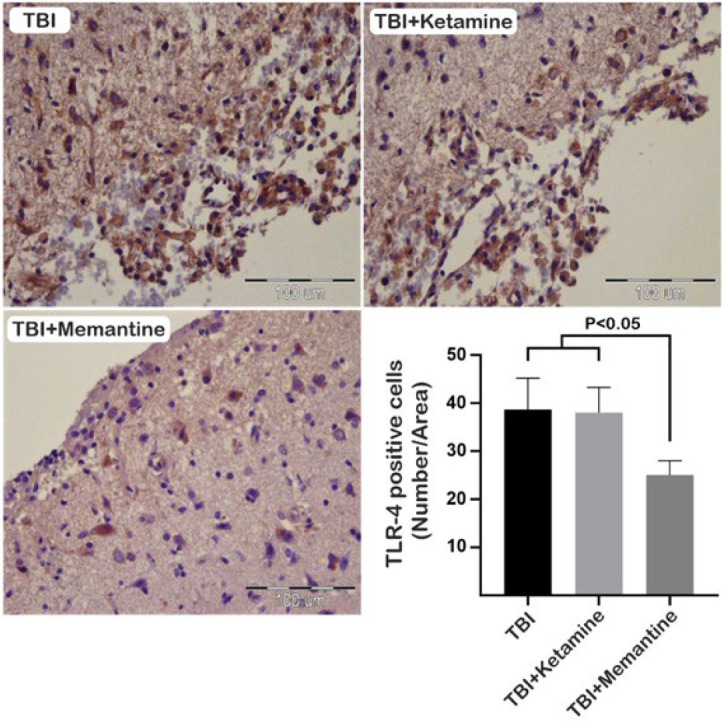
Effect of ketamine and memantine on the expression of TLR-4 following TBI in rats

**Figure 5:**
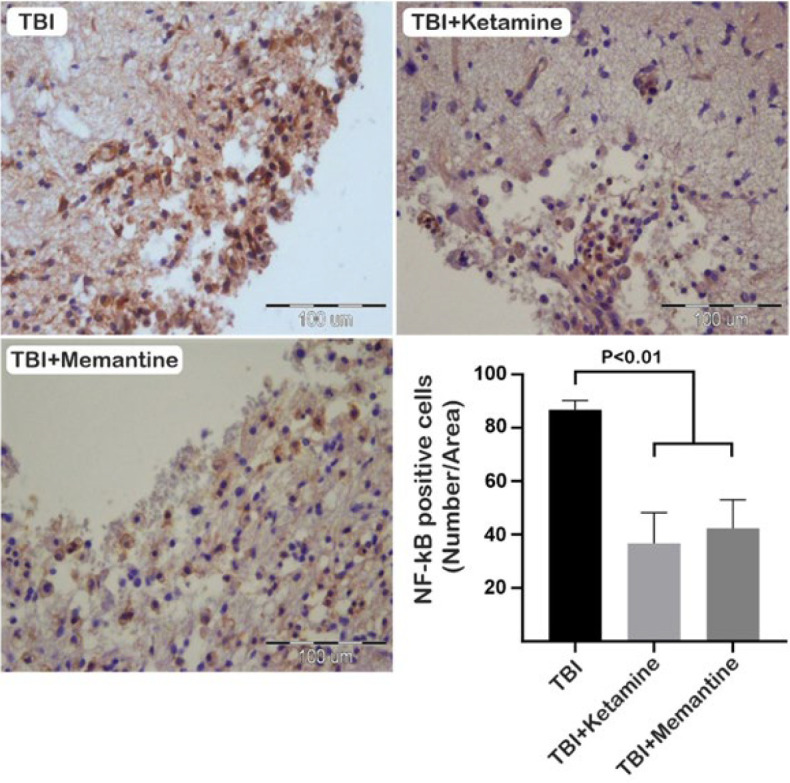
Effect of ketamine and memantine on the expression of NF-κB following TBI in rats

Our study looked at the TLR4/NF-κB pathway as a molecular mechanism involved in neuroinflammation following TBI. Our results show that the number of TLR-4 positive cells was significantly reduced in the TBI+Memantine group compared to the TBI+ketamine and TBI groups at the injury site seven days after TBI (Figure 4). Additionally, immunohistochemical staining of NF-κB, a downstream molecule of TLR-4, showed that the number of NF-κB positive cells increased after injury. However, treatment with ketamine and memantine significantly decreased the expression of NF-κB when compared to the TBI group 7 days after injury (Figure 5).

(A, C) Assessment of anti-oxidant defense (Thiol and SOD) and lipid peroxidation (MDA) levels in the brain tissue 24 hr after TBI. Ketamine and memantine significantly promote an antioxidant defense system. In addition, memantine reduces the level of MDA in animals. Data are shown as the mean ± SD (n = 6/group).

TBI: Traumatic brain injury

Iba-1 was used for label of microglia via IHC. The statistical analysis of the number of Iba-1 positive cells was not different in all experimental groups. Data are shown as the mean ± SD (n = 5/group). TBI: Traumatic brain injury

IHC was used to detect GFAP-positive cells in the lesion site seven days after injury. The bar graphs show that memantine significantly reduced the number of GFAP-positive cells compared to the TBI group. Data are shown as the mean ± SD (n = 5/group).

TBI: Traumatic brain injury

TLR-4 is one of the primary receptors that increase after TBI. The image shows the expression of TLR-4 within the lesion site seven days after injury. The mean number of TLR-4 positive cells in TBI+memantine markedly reduced compared to TBI+ketamine and TBI groups in the lesion site. Data are shown as the mean ± SD (n = 5/group). TBI: Traumatic brain injury

The image showed the mean number of NF-κB in the lesion site seven days after TBI. The bar graph presents a significantly lower expression of NF-κB in the TBI+ ketamine and TBI+ memantine compared to the TBI group. Data are shown as the mean ± SD (n = 5/group). TBI: Traumatic brain injury

## Discussion

Our study aimed to investigate the potential of ketamine and memantine as NMDA receptor antagonists in mitigating the effects of TBI. Our results showed that these drugs have promising effects on enhancing anti-oxidant capacity and modulating the neuroinflammatory signaling pathway. Oxidative stress, which results from an imbalance between anti-oxidants and ROS/reactive nitrogen species (RNS), is one of the primary mechanisms responsible for secondary damage after TBI (28, 29). Superoxide (O^2-^) is the most common type of free radical that promotes the formation of ROS/RNS (30). NMDA receptor activation in injured cells can promote oxidative stress by releasing superoxide into the extracellular space through NMDA receptor-induced activation of NOX2 (31). Ketamine restored the level of MDA, an oxidative stress marker, in the brain to near the level of the control group. Ketamine can also exert neuroprotection against TBI by activating the Nrf2 pathway and its downstream proteins (32). Furthermore, memantine has shown promise in modulating oxidative stress by stimulating the SOD1-relevant anti-oxidant defense system in experimental autoimmune encephalomyelitis (EAE), a model for multiple sclerosis. It partially regulates amino acid homeostasis, suggesting potential therapeutic benefits in MS (33). Our study found that ketamine did not alter MDA levels in the injured cortex but increased SOD levels and total thiol. Memantine also increased SOD and total thiol levels while reducing MDA concentrations. Based on this data, memantine is significantly more likely to improve oxidative stress outcomes than ketamine. This is because memantine can more effectively inhibit the imbalance between anti-oxidant and oxidant systems than ketamine. The activation of glial cells characterizes neuroinflammation in TBI, the release of inflammatory mediators, and the recruitment of peripheral immune cells to the injury site (34). Neuroinflammation during TBI alters the phenotype of glial cells from a neuroprotective to a classical phenotype, resulting in the overproduction of neurotoxic factors (35-37). Proinflammatory cytokines may promote degeneration by NMDA-induced calcium influx through their effect on glial cells (38). Ketamine and memantine can potentially decrease glutamate excitotoxicity and alleviate secondary insult after TBI (39, 40). However, the effects of ketamine on inflammation remain controversial (40-42). In anesthetic doses, ketamine has an anti-inflammatory effect, but in sub-anesthetic doses, its impact on TBI-induced inflammation is minimal (41). Our study found that ketamine has a minor impact on inflammatory signaling pathways via down-regulation of NF-κB in the injury site and did not strongly affect glial cell activation. On the other hand, memantine may improve the pathophysiology in the acute phase of TBI by inhibiting the expression of NR2B, the extrasynaptic type of NMDA receptors (43). Previous studies have shown that memantine does not significantly reduce the number of Iba-1-positive cells after TBI (14, 44). However, memantine reduced GFAP-positive cells in the injured brain (44). These studies are based on our findings, where the administration of memantine did not alter the expression of Iba-1; however, it reduced the expression of GFAP on the injury site. Moreover, memantine had a notable effect on the inflammatory signaling pathway by modulating the expression of TLR-4 and NF-kB on the injury site.

## Conclusion

In this study, we investigated the potential role of ketamine and memantine as NMDA receptor antagonists in mitigating the effects of TBI. The results suggest that both drugs can enhance anti-oxidant capacity and positively modulate the neuroinflammatory signaling pathway. Further studies are warranted to focus on exploring the efficacy of combining ketamine and memantine in investigating the long-term effects of these drugs on TBI-induced neuroinflammation.

## Data Availability

The data supporting this study’s findings are available upon request from the corresponding author.

## Declaration

We acknowledge the use of Grammarly to improve the language and readability.
